# Diffusion Tensor Imaging for Ruptured Cerebral Arteriovenous Malformation

**DOI:** 10.7759/cureus.1721

**Published:** 2017-09-28

**Authors:** Muhammad Waqas, Ayesha Siddiqui, Fatima Mubarak, Syed Ather Enam

**Affiliations:** 1 Surgery, The Aga Khan University; 2 Radiology, The Aga Khan University

**Keywords:** arteriovenous malformation, diffusion tensor imaging (dti), fractional anisotropy (fa)

## Abstract

Non-ruptured arteriovenous malformations (AVMs) rarely cause tract disruption. Few studies have described how ruptured AVMs influence white matter (WM) tract morphology. We reviewed consecutive AVM cases treated at a tertiary care hospital where diffusion tensor imaging (DTI) tractography was obtained preoperatively. DTI was performed using the Synaptive Plan (Synaptive Medical Inc., Toronto, Canada). Quality control was performed by clinical application specialist. Perinidal fractional anisotropy (FA) value of corticospinal tracts (CST) was obtained. A reference FA value was obtained from the corresponding area on the contralateral side. Images were evaluated by a consultant neuroradiologist. Radiological findings were correlated with clinical findings. White matter morphology was described by a consultant neuroradiologist.

All three cases included in the study had a history of haemorrhage in the past. Two patients had disruption of CST and presented with a significant neurological deficit. In one patient FA value of CST around the nidus was comparable to the contralateral side and did not show any neurological deficit. DTI integrated neuronavigation was used to plan the trajectory and complete resection of the AVM with excellent postoperative recovery.

## Introduction

Diffusion tensor imaging (DTI) is a non-invasive imaging modality that determines the spatial distribution of the diffusion of water molecules. It provides a three-dimensional image of the white matter (WM) tracts. The extent of white matter damage can be measured by fractional anisotropy (FA) across the tracts through DTI tractography [1].

FA measures the preferential direction of the diffusion of water molecules [1]. The information on the structural integrity of the WM tracts is estimated by different intensities on DTI color map. WM tracts close to the nidus as measured by means of FA value may be less apparent than the corresponding normal tract. For brain tumors lying near the WM fibers, FA can determine the viability of those tracts for surgical purposes.

Arteriovenous malformation is the congenitally localized anomalous accumulation of dilated arteries and veins which lack a capillary network subsequently creating a direct channel between the cerebral arteries and veins, risking rupture [2]. Few studies have described the morphology of WM tracts for cases of non-ruptured arteriovenous malformations (AVMs). Although intracerebral hemorrhage in itself can cause significant tract disruption [3], the effect of AVM rupture on tracts has not been explored extensively.

The purpose of this study was to describe the morphology of white tracts on the basis of DTI. We then described the clinical correlation of morphological information with the clinical picture. We utilized DTI to evaluate the integrity and spatial relationship of the tracts to the lesion and chose a safe surgical corridor in three patients by using it for neuronavigation.

## Case presentation

Technical details 

DTI studies were performed using the Synaptive DTI protocol (Synaptive Medical, Inc., Toronto, Canada) on Siemens© 3 Tesla machine (Siemens Healthcare, Erlangen, Germany). The tractography was generated using the BrightMatter™ Plan (Synaptive Medical, Inc., Toronto, Canada). The protocol required a minimum of 20 gradient directions preferred +1 baseline B0 scan. B0 is an image of the anatomy that takes into account tissue signals and contrasts in the absence of diffusion gradients. B value of 1,000 was preferred. Slices of 2 mm thickness were obtained with gap/spacing 0.0 mm, square matrix 128 x 128. A DTI scan was completed prior to contrast injections. All the images were evaluated for their quality using a quality control (QC) algorithm. The QC was performed by the Synaptive ImageDrive™ (Synaptive Medical, Inc., Toronto, Canada) by a Synaptive clinical application specialist. The protocol was used as part of the BrightMatter™ Guide (navigation) and BrightMatter™ Drive (robotic arm) technology (Synaptive Medical, Inc., Toronto, Canada). FA values were calculated by selecting a region of interest (ROI) close to the nidus in corticospinal tracts (CST) on FA map. Corresponding FA value was obtained from the identical area on the uninvolved side by estimating left-right asymmetry. Selection of ROI and calculation of FA value was performed by a consultant neuroradiologist. Selected cases were studied in detail using the hospital electronic database and medical records. Demographic, clinical, and radiological variables were recorded and described. A correlation between FA values, white matter integrity, and neurological deficits was made. Surgical resection was aided by neuronavigation with DTI and CT angiograms merged by advanced options on the BrightMatter™ Guide for all the cases.

Case 1

**Figure 1:**
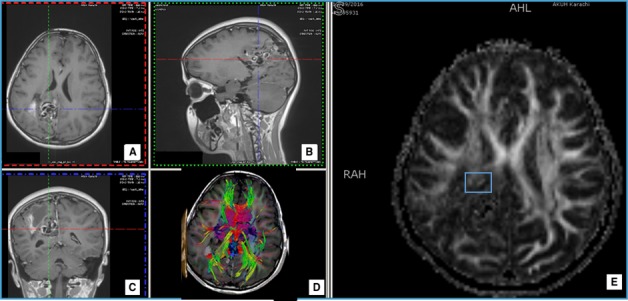
Case 1 T1 post-contrast images: axial (A), sagittal (B), (C) and (D) large nidus of serpiginous vascular channels in the right parietal region showing dilated draining veins with a venous aneurysm near the midline representing a complex arteriovenous malformation (AVM). Region of interest for fractional anistropy (FA) value has been highlighted on the FA map (E).

A 12-year-old girl presented with left-sided hemiparesis following a right frontoparietal hemorrhage two months prior. On examination, she was fully awake, alert, and oriented. She had pyramidal weakness in her left upper and lower limbs of grade 4/5. Magnetic resonance imaging (MRI) of the brain with contrast demonstrated a right parietal intraparenchymal hemorrhage with maturation changes suggested by a hemosiderin rim. The lesion appeared hyperintense on T1 and T2 images with central diffusion restriction. It measured 65 x 48 x 37 mm in the anteroposterior, craniocaudal, and transverse dimensions (Figure 1). In comparison with a previous computed tomography (CT) scan, a slight interval increase in size was noticed; however, the perilesional edema and midline shift had resolved. A large nidus of serpiginous vascular channels in the right parietal region showed dilated draining veins with a venous aneurysm near the midline representing a complex AVM. The feeding artery appeared to arise from the left posterior cerebral artery with drainage into the superior sagittal sinus. Gliosis and encephalomalacia were identified in the right parietal lobe with parenchymal volume loss secondary to a vascular anomaly.

Tractography showed fibers of the superior and posterior regions of the corona radiata, superior longitudinal fasciculus, forceps major, and tapetum were disrupted with an FA value of 181.4 as compared to the contralateral normal FA value of 494.8 (Figure 1). The AVM was Spetzler-Martin grade 4. The images of the AVM are shown in Figure 1. Following the imaging studies, a neuronavigation-guided excision was performed. Tractography was incorporated to the navigation images on the BrightMatter™ Guide. Resection was assisted by microscope and video microscope (BrightMatter™ Drive). The patient showed a good recovery with subtle residual weakness.

Case 2

**Figure 2:**
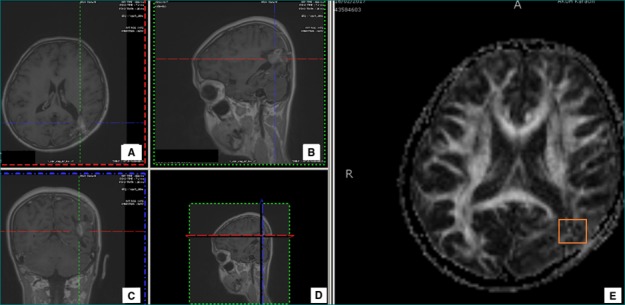
Case 2 T1 contrast-enhanced images with axial (A), sagittal (B), and coronal (C) sections showing an arteriovenous malformation (AVM) in the left parieto-occipital region. Encephalomalacic changes and ex vacuo dilatation of the occipital horn of the left lateral ventricle in the corresponding area was noticed. Fractional anisotropy (FA) map shows the region of interest for the calculation of FA value (D, E)

A 15-year-old boy presented with a three-month-old left parietal intraparenchymal hemorrhage. A CT angiogram showed a left parietal AVM (Spetzler-Martin grade 2) (Figure 2). The clot was evacuated three months previously, and on presentation, the patient had no significant neurological deficit.

The AVM was fed by a branch of the left posterior cerebral artery and drained by two vessels into the left transverse and superior sagittal sinus. Encephalomalacic changes and ex vacuo dilatation of the occipital horn of the left lateral ventricle in the corresponding area were noticed.

On DTI tractography, the left inferior longitudinal fasciculus, forceps major, posterior region of the corona radiata, and the superior longitudinal fasciculus were poorly visualized without any obvious disruption (Figure 2). The FA value was reduced with abnormal hues on the directional map. The FA value was 321 as compared to contralateral normal of 483.

The AVM excision was performed with a stable postoperative recovery. At his five-month follow-up, his headaches had subsided and he had resumed daily school activities.

Case 3

A 26-year-old male presented with right-sided numbness and intermittent mild to moderate headache for one year. He had an episode of intraparenchymal hemorrhage a year previously with a severe headache and transient loss of consciousness. An AVM (Spetzler-Martin grade 3) was found.

**Figure 3:**
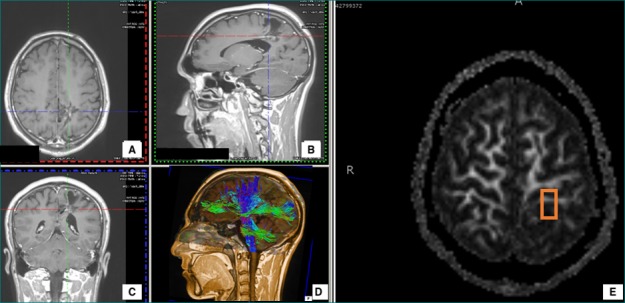
Case 3 T1 contrast-enhanced images with axial (A), sagittal (B), and coronal (C) sections showing an arteriovenous malformation (AVM) in the left parietal region. Image D is showing various tracts in relation to the AVM. Encephalomalacic changes and ex vacuo dilatation of the occipital horn of the left lateral ventricle in the corresponding area was noticed. Fractional anistropy (FA) map (E) shows the region of interest for the calculation of the FA value.

On DTI tractography, fibers of the posterior corona radiata and the posterior part of the medial limb of the superior longitudinal fasciculus were disrupted with abnormal color hues on a colored map (Figure 3). An FA value of 324.6 was calculated as compared to contralateral normal of 578.9. Surgical removal of the AVM was carried out successfully with no complications postoperatively. Follow-up eight months after surgery revealed a reduction in symptoms and an improved quality of life.

## Discussion

In this report, we have presented three cases of AVMs with previous rupture. We utilized classifications system introduced by Witwer, et al. to describe the morphology of WM tracts [4]. Two of our cases had perinidal disruption of CST while the other had tract edema. This correlates well with the clinical picture as the cases with tract disruption had a significant neurological deficit at the time of presentation. The clinical correlation of WM morphology and neurological deficits have been described in a few retrospective studies. In 2012, Ellis, et al. described three pediatric cases of ruptured AVM [5]. One of these children with severe motor deficits showed tract reduction and displacement. The morphological description of the WM tracts provided by the study was, however, arbitrary. Another study evaluating WM tract morphology found no disruption of CST in cases of unruptured AVM [6]. This was reflected in the clinical picture, which did not show any neurological deficit. Four of the ruptured cases of AVM had some tract disruption with neurological symptoms. Tract disruption thus observed may be due to hemorrhage and not the AVM nidus. DTI has been employed before to AVMs to observe the interaction between the lesion and the white matter tracts. In 2005, Yamada, et al. described the utility of DTI tractography in identifying eloquent cortices, as well as the communication of the white matter fibers with the nidi or the drainage vessels of the AVM [7].

Our results are congruent with the aforementioned studies, showing the quantitative analyses to be more reflective of the symptomatology of the patients than the appearance of the tracts. The FA value of Patient 1 was around three times lower compared to the normal contralateral, with Patient 3 having an FA value two times lower than its contralateral unaffected side, and both patients had neurological deficits. Patient 2 had no neurological symptoms and did not appear to have a significantly low FA value in comparison to the normal side.

The lack of visualization of the corticospinal WM tracts has been shown to not necessarily be correlated with associated symptoms, particularly in hemorrhage [6]. In our case series, patients who were symptomatic exhibited interrupted or non-visualized fibers, while the patient who was asymptomatic had no evident disruption of tracts. As all patients underwent hemorrhage, the comparison with a non-hemorrhagic AVM could not be established. However, it can be questioned whether the time from a hemorrhagic event is significant in affecting the WM fibers as well as the FA value since Patient 1 had the most recent history of bleeding with the greatest difference in FA value and had disruption of fibers. Besides serving as a presurgical assessment tool, DTI tractography has been emerging in the field of radiation therapy. It has proven to be a vital tool in determining the dosage and preventing morbidity for stereotactic radiosurgery in AVM management [8]. The Spetzler-Martin scale has long been used to predict the outcome of surgery. It might be useful to integrate DTI information into preexisting prognostic tools.

The surgical resection of AVM follows the “all or nothing” principle. Microsurgical resection is relatively superior to stereotactic radiosurgery and endovascular embolization wherever possible [2]. DTI, especially in cases of ruptured AVMs, can guide prognosis and counseling of families. DTI neuronavigation as utilized in this study using the Synaptive BrightMatter™ Guide can also help in selecting a safe surgical corridor, making sure that a complete excision is performed without risking WM tract integrity. Use of DTI for the selection of a surgical corridor is well-described [9]. DTI is integrated with various MR neuronavigations. Tract morphology and relationship of the lesion to CSTs are carefully studied to choose a safe corridor. Although incomplete resection is not an option, it is possible to use DTI to perform a resection.

The study has a limited number of cases; however, it provides an important perspective on the role of DTI in the management of AVM. We have utilized a standardized and well-described nomenclature to describe WM tract morphology. This is the first study to describe the use of the Synaptive DTI protocol and Guide in the understanding of WM. We recommend more prospective studies on the topic for improved understanding of the topic.

## Conclusions

Ruptured AVMs can result in non-visualization and disruption of tracts around the nidus. Tract morphology around the nidus correlates with the neurological deficits in a patient and may be used to guide surgical planning, prognosis, and outcomes.
